# Women who use drugs: engagement in practices of harm reduction care

**DOI:** 10.1186/s12954-023-00775-0

**Published:** 2023-04-13

**Authors:** Tamar Austin, Jennifer Lavalley, Sylvia Parusel, Alexandra B. Collins, Michelle Olding, Jade Boyd

**Affiliations:** 1 Birth Place Lab, UBC Midwifery, Faculty of Medicine, University Boulevard, Vancouver, BC 3302-5950 Canada; 2 British Columbia Centre On Substance Use, St. Paul’s Hospital, 400-1045 Howe Street, Vancouver, BC V6Z 2A9 Canada; 3 Interdisciplinary Studies Graduate Program, University of British Columbia, Vancouver, BC Canada; 4 Department of Epidemiology, Brown University School of Public Health, Providence, RI USA; 5 Department of Medicine, University of British Columbia, St. Paul’s Hospital, Burrard Street, Vancouver, BC 608-1081 Canada

**Keywords:** Women, Substance use, Harm reduction, Care, Overdose, Gendered labour, Canada, Care ethics

## Abstract

**Background:**

Harm reduction services that employ or are operated by people who use drugs are an effective means of mitigating overdose risks and other drug-related harms. However, stereotypes portraying people who use criminalized drugs as incapable caregivers persist. This is especially true for women who use drugs, and to a greater extent racialized women, who are characterized as having diverged from traditional ideals of womanhood as a result of drug-user stigma and the intersections of gender- and class-based and racist stereotypes. In an effort to identify and understand how women who use drugs practise care through harm reduction, we explored the experiences of women accessing a low-threshold supervised consumption site exclusively for women (transgender and non-binary inclusive) in Vancouver, Canada.

**Methods:**

Data were drawn from research conducted from May 2017 to June 2018 exploring women’s experiences accessing the supervised consumption site during an overdose crisis. Data included forty-five semistructured interviews with women recruited from the site, analysed thematically to explore practices of care through harm reduction.

**Findings:**

Participants reported engaging in both formal and informal care. Acts of care included interventions that both aligned with and deviated from conventional understandings of care practices, including overdose reversal and education, overdose supervision/care, and assisted injection.

**Conclusion:**

The boundary between formal and informal harm reduction care is fluid. Women who use drugs engage in harm reduction across these borders with acts of care that align with or fill the gaps in current harm reduction services in order to meet the needs of drug-using communities, challenging negative stereotypes of women who use drugs. However, these caregiving practices can increase risks to care providers’ physical, mental, and emotional health and wellness. Increased financial, social, and institutional supports, including safer supply, assisted injection, and community resources, are needed to better support women as they continue to engage in harm reduction care.

## Background

Across Canada and the USA, communities are contending with an overdose epidemic driven by fentanyl-adulterated drug supplies and exacerbated by prohibitionist drug policies and structural barriers. Between January 2021 and January 2022, the USA reported an estimated 77,607 opioid overdose deaths [1]. Between January 2021 and December 2021, Canada reported 7,560 opioid-related deaths [2]. Within Canada, British Columbia (BC) continues to be one of the most severely impacted provinces, reporting 2,262 opioid-related drug deaths in 2021 and holding the highest rate of overdose deaths in the country [2]. Given these conditions, harm reduction strategies in Canada have been implemented and scaled up in recent years with the aim of mitigating risks and harms related to the criminalization of drug use, including overdoses.

Although there is no universally accepted definition of harm reduction, it can be understood as policies, programmes, or practices intended to mitigate health, social, and legal consequences stemming from drug use, drug policies, and laws [3]. In response to the increasingly toxic drug supply in BC, numerous low-barrier supervised consumption sites have emerged, called overdose prevention sites (OPS) [4, 5]. Unlike their predecessors, supervised consumption sites (SCS), OPS are not legally obliged to hire a health-care practitioner to supervise drug consumption [4] and rely heavily on the paid or voluntary care of people with lived experience (PWLE) of substance use as “peer workers” [6].

In BC, the employment of PWLE in harm reduction services and organizations scaled up considerably in response to the overdose epidemic [7, 8]. Previous research indicates the harm reduction labour of PWLE can foster an environment of inclusivity, provide a sense of community, and facilitate feelings of comfort for clients [7, 9, 10]. Additionally, PWLE possess an intricate knowledge of the structural vulnerabilities and risks routinely navigated by people who use drugs and can therefore facilitate engagement with hard-to-reach populations [7, 9, 10].

Despite the positive impacts of peer harm reduction workers at the individual and community level, peer workers contend with challenges as a result of institutional, organizational, and intersecting socio-structural barriers. Peer work can be precarious, with sporadic and intermittent scheduling [6, 11, 12] and often inequitable and insufficient compensation in comparison to other support and front-line workers [7, 10, 12, 13]. Further, experiences of workplace discrimination are persistent and serve to reinforce the lack of recognition and respect PWLE receive due to a history of drug use or a lack of formal certification [6, 7, 10, 12].

While PWLE can find harm reduction work fulfilling, it can negatively impact their emotional and mental health. Their continued navigation of socio-structural barriers (i.e. housing instability, poverty, criminalization, stigma) while experiencing personal loss and workplace trauma (i.e. unprecedented overdose deaths) can engender feelings of burnout [6, 7, 10, 12]. These factors can have a cumulative effect where structural barriers leave them unable to connect with resources that other support and front-line workers may access in similar circumstances [7]. As a result, peer workers may decrease their work hours or withdraw from their positions due to a combination of emotional burnout, workplace inequities, and a lack of access to support services [7, 12].

At the same time, the legitimacy of the care they provide is perpetually challenged by individuals, institutions, and the state [12, 14]. Stereotypes of people who use illegal drugs as selfish, uncaring, and intrinsically criminal are pervasive [15]. Media depictions perpetuate and reinforce negative images of people who use drugs, and prohibitionist drug policies and harsh anti-drug laws mirror [16] and reinforce these stereotypes [17]. Such characterizations ignore the multiple ways people who use drugs engage in providing care and support within their communities and interpersonal relationships.

While what constitutes “care” remains a contested concept, its parameters have expanded in recent times. Drawing from Foucault’s ethics of care, critical scholars have articulated that care is best understood within the relational context in which it is practised [18]. Feminist-informed care ethics, for example, are concerned with fully understanding and meeting the needs of others [19, 20] and posit that care is dependent on the relationships of the care provider and receiver [19, 21, 22], the social, cultural, and environmental contexts in which care occurs [21], and the power dynamics which govern the relationship between the provider and receiver [19, 20]. Feminist care ethics call for care providers to “[know] other people without objectifying them”, which is only made possible by listening to the care receiver [19], p. 846). Further, feminist care ethics require care providers to be flexible in order to meet the specific needs of the care receiver, recognizing that this may call for care providers to work outside the established institutions, policies, and frameworks when they do not appropriately satisfy the needs of those they are meant to serve [22].

For people who use drugs, engagement in care is governed by multiple factors, including drug laws and regulations [17, 23], an individual’s built environment and life conditions [18, 24], and gender and relationship dynamics [23, 25]. People who use drugs apply their own “ethos of care” throughout their practices of harm reduction [23], which are focused on meeting the needs of the community they exist in. Thus, harm reduction care practised by people who use drugs can be understood as the labour they engage in for themselves and others, within a drug-prohibitionist environment, while simultaneously navigating institutional, structural, and social barriers.

These harm reduction-based acts of care can diverge from normative, abstinent-based conceptions of care, which focus on the reduction of drug consumption. Reconceptualized acts of care within drug-using communities can present as mitigation of pain [14], decreasing risks associated with drug procurement [9], the negotiation of injection order between intimate partners framed by disease transmission and gender [23, 25], assisted injection [26, 27], or the sharing, diversion and consumption of drugs [18, 24, 28]. Drug prohibition, stigmatizing policies and laws [9, 17], and normative views of people who use drugs [9, 14] negatively impact the forms of drug-related care people who use drugs are able to provide and increase risks for specific sub-groups. This may be particularly true for women who use drugs (WWUD), as they experience drug-related risks differently than men, and gendered social expectations influence women’s engagement in care [29–31].

Institutions, societal practices, and interpersonal relationships perpetuate, maintain, and reinforce gender norms [32, 33], which regulate and stereotype care as women’s work. In Canada, women disproportionately engage in unpaid labour (i.e. housework and caregiving) compared to men, despite increased participation in the paid labour force [34] and are overrepresented in care-based sectors such as teaching, nursing, and social work [35]. However, WWUD, especially Black, Indigenous, racialized, and socio-economically marginalized women, are excluded from narratives of gendered care. In contrast, they are often perceived as unfit caregivers, depicted as having deviated from traditional notions of womanhood and caregiving; and viewed as being incapable of filling the roles of nurturers, caregivers, or mothers due to their drug use [15, 36]. Negative perceptions of WWUD are further impacted by intersecting racist narratives, resulting in policies (e.g. child apprehension) that disproportionately negatively impact Black and Indigenous women [37–40]. Thus, Indigenous and Black women who use drugs must contend with the intersecting effects of racialization and gendered drug-user stigma while attempting to fulfil caregiving roles.

Gender inequities, racism, and drug-use-related discrimination intersect to shape WWUD’s experiences of providing harm reduction care, yet there remains a dearth of literature on the subject. Our paper aims to address this gap by utilizing a feminist-informed ethics of care framework to analyse practices of care performed by socio-economically marginalized WWUD. A feminist care ethics approach is useful as it focuses attention on how women’s care practices are shaped by gendered intersecting power relations and constructs, a guiding question for this study. Understanding the ways in which WWUD are uniquely responsibilized for harm reduction care practices in the context of structural inequality and prohibition has important implications for addressing women’s emotional, physical, mental, and financial well-being.

## Methods

This paper draws from ethnographic research conducted at SisterSpace, a women-only and transgender-inclusive low-barrier overdose prevention site (OPS) located in the Downtown Eastside of Vancouver, British Columbia. As a methodology, ethnography is focused on understanding people’s actions within the contexts they exist in [41]. Ethnography utilizes a number of methods to understand the perspectives of those being studied, including interviews and observations [41], both of which were employed in this study. Data were collected between May 2017 and June 2018, exploring women’s experiences accessing SisterSpace during an overdose crisis. Data included forty-five semistructured interviews with women recruited from the site, accompanied by approximately 100 h of ethnographic fieldwork conducted on-site and in the surrounding area by the senior researcher. Study participants were recruited from the OPS by the senior author and peer research assistants (local community members with experience of criminalized substance use trained in research activities), and interviews took place at a nearby research office. A collaboratively designed interview guide (drawing on the input of a community advisory board) facilitated conversations around a range of topics on women’s drug use and overdose prevention. Women who consented took part in forty-five- to sixty-minute interviews and were compensated with Can$30 honoraria for their time. Interviews were audio recorded and transcribed verbatim. All identifying information was removed and participants were assigned pseudonyms via an online name generator. The study received ethical approval from the University of British Columbia/Providence Health Care Research Ethics Board.

Transcripts were uploaded into qualitative data analysis software NVivo and thematically analysed. A coding meeting was held to identify initial themes related to gender, drug use, and overdose experiences. Emergent themes [42], including those that explored participants’ engagement in formal and informal acts of harm reduction care within their communities, were developed during analysis. Throughout the analysis process, focus was given to the presence of gendered norms and power dynamics within women’s interpersonal relationships. The prioritization of these themes allowed us to explore the ways in which WWUD’s acts of care meet the needs of the communities they exist in. We further drew on an approach grounded in feminist care ethics, which centres the importance of relationships in caring labour, acknowledges the role power dynamics play in caring relationships [20], and recognizes women’s ability to be flexible and work around laws and policies [22] that may have adverse impacts on people who use drugs as harm reduction fluidly moves between the private and public spheres.

### Sample population characteristics

**Fig. 1:**
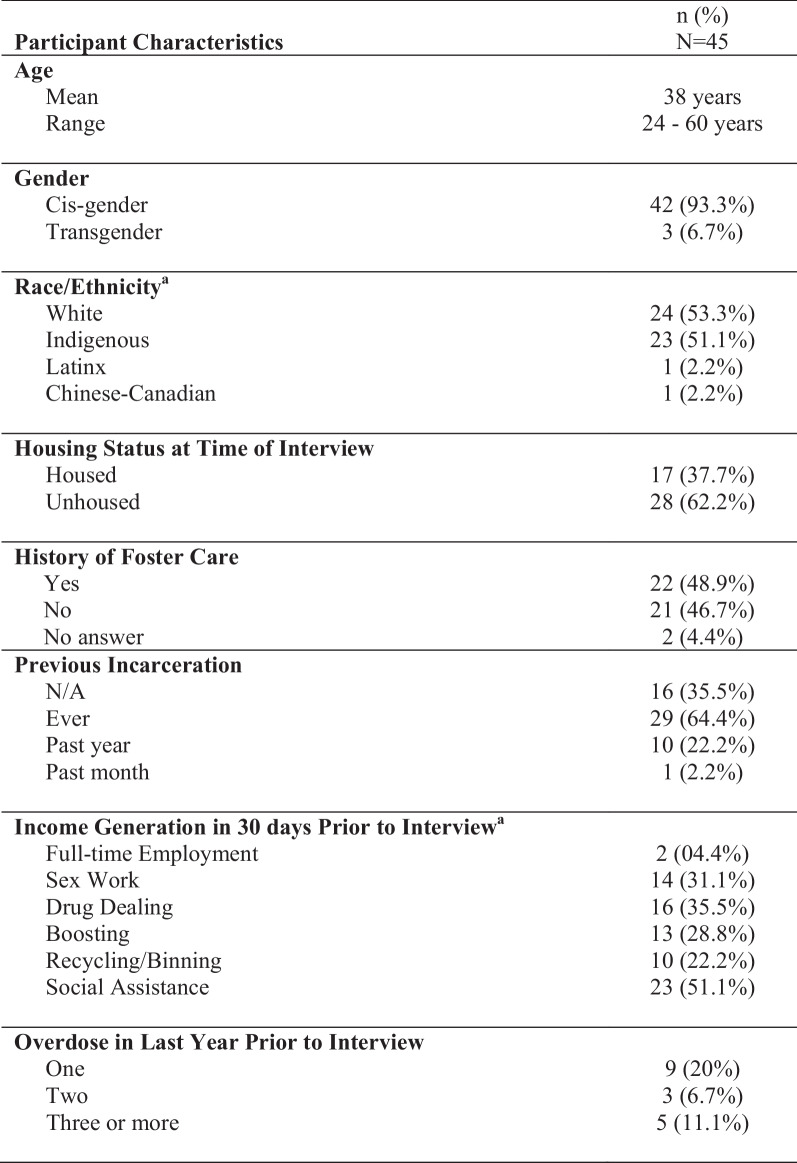
^a^Participants could select more than one

A total of 45 women participated in this study, averaging 38 years of age (range 24–60 years). The majority of women identified as white (*n* = 23) or Indigenous (*n* = 22). Several participants identified as more than one race, including East/Southeast Asian (*n* = 1), Latinx (*n* = 1), Chinese (*n* = 1), and other (*n* = 1). Indigenous women were overrepresented in this study, accounting for 49% of the study population, yet comprising 4.9% and 2.3% of the total population in Canada and Vancouver, respectively [43]. Cisgender women comprised the majority of participants (*n* = 42), while the remaining women identified as transgender (*n* = 3). Heroin (*n* = 38), crystal methamphetamine (*n* = 30), and fentanyl (*n* = 20) were the most commonly reported drugs used amongst study participants within the thirty days leading up to their interviews, and the majority of participants identified as polysubstance users (*n* = 41). Most women (*n* = 28) were unhoused at the time of the interview. Fifteen women reported being in a relationship at the time of the interview. See Fig. 1.

### Findings

The majority of participants (*n* = 28) discussed their experiences engaging in practices of formal and informal harm reduction care within their interpersonal relationships and wider community, while navigating their own drug use. The following sections expound upon the diverse forms of harm reduction care practised by participants, and how these practices impacted their physical, emotional, and mental well-being.

#### Formal care: overdose reversal and education

Several women in our study described navigating their roles as paid or voluntary peer workers in formal harm reduction settings, such as OPS and supportive housing with harm reduction interventions. Tasks included, but were not limited to, janitorial services (e.g. cleaning), domestic tasks (e.g. making coffee and providing snacks), or harm reduction services (e.g. responding to overdoses, educating on safer drug use). Sometimes, as we also observed, women performed all three of the aforementioned roles simultaneously. For example, “Rose”, a 35-year-old Indigenous woman, described the multiple forms of care she performed within the confines of her formally recognized peer position:

*The peers were doing the intakes. We clean up after they use [drugs]. You know, we just watch them, make sure they don’t pocket the med [medications], and… I clean up after them and we got a coffee thing and I make them get their coffee. […] You’re just walking around and… or sitting, just watching everyone, and when they’re done with their table you clean, with a Cavi[disinfecting] wipe, and give the next number and the person who’s in line. And then mop and sweep and, you know, make sure everyone’s safe… We respond to overdoses in the alleys and in… we did one in [a community centre] bathroom.*

Rose’s harm reduction care included the supervision of clients as they consumed drugs, the collection of used subdermal syringes, and the distribution of sanitary harm reduction supplies. Other participants provided similar in-depth descriptions of their regular responsibilities within a formal workplace. “Lori”, a 49-year-old white woman, described her role as more complex than how she felt it was commonly perceived:

*[Some people think] you sit around and wait for people to fucking get their high and then you wait for them to die. You know, it’s just like, wow. But no, it’s educating them to use properly. You know, to change the needles, educate them on how to find veins. Oh yeah. It’s awesome.*

Notably, Lori’s work in harm reduction extended beyond overdose interventions, into the role of a peer educator, imparting drug-use knowledge that may not otherwise be available to her clients, with potential to decrease injection-related risks.

Lori explained that she associated her work with “keeping people safe and… giving back to the community”, and she appeared to derive a sense of pride from the level of care she invested into her community. However, formal peer work also has negatives. For example, “Marisol”, a 30-year-old Indigenous woman, disclosed that she had consistently experienced difficulties obtaining payment from her place of work.

Although these descriptions of women’s peer work were valuable, our observations and further discussion revealed that harm reduction care was fluid, extending beyond the confines of formal peer work into informal spaces and acts of care.

#### Informal care: overdose supervision/care

Several participants reported engaging in harm reduction care for family members, friends, and strangers outside of formal work environments. For these women, care could present as providing overdose intervention care and/or assisted injection. Attending to overdoses via the administration of naloxone, cardiopulmonary resuscitation (CPR), or physical stimulation was a recurring event in participants’ lives. “Sam”, a 32-year-old Indigenous woman, recounted administering Naloxone to her cousins on two different occasions:

*My cousin OD’d three times in my bathroom, and the first time he just came out of treatment… I was making dinner and I kind of heard him… I don’t know. He fell down, but he didn’t… I think his head hit the… hit the potty and I could hear like just slightly, and I went and knocked on the door and he wasn’t answering me, and I called my boyfriend and said I can’t get him... I had to kick the door in and break the… break the door. […] And the second time, yeah, yeah, each time I had to Narcan him three times… My other cousin, I had to Narcan him on the street… And it’s a good thing that someone had a Narcan kit there. It was actually someone passing by and none of us had… and it took like thirty minutes for the ambulance to get there…*

Sam’s experience was not unique. Just under half of the study participants shared experiences of performing overdose prevention in an informal capacity and in high-risk environments, including alleyways, parks, and other areas where risk of overdose was higher as a result of the unsupervised nature of outdoor drug consumption.

Study participants explained that attending to overdoses triggered a range of negative emotions. “Dawn”, a 44-year-old white woman, shared that the experience was “scary… when you see someone’s life being drawn out of them, it scares you. It wakes you up”. Some participants, such as “Max”, a 30-year-old white woman, showed discomfort in discussing occasions on which they would attended to overdose events. When asked about one such experience, Max stated, “[it’s] in the past. I’d like to leave it there”. “Rhonda”, a 40-year-old white woman, described a trajectory in which attending to overdoses went from a traumatic new experience to a numbing routine:

*The first time I ever saved a life it was like, like I had to go puke after. It was just like oh my god. Then after a while it was like nothing… I mean it’s still obviously intense because at the moment the person is pretty much dead or not breathing… but it’s honestly, it’s so routine now. It’s crazy. People are very, down here especially, very immune to it. Just numb. Somebody goes down, people are like “Narcan!”*

Overdose interventions further appeared to be the primary form of harm reduction care participants invested into their intimate relationships. “Mae”, a 47-year-old Indigenous woman*,* recalled intervening multiple times in her partner’s overdoses, stating, “My boyfriend has low… very low tolerance. He OD’d [overdosed] twenty-one times… I responded [to] a lot of them”. Another participant, “Esme”, a 34-year-old Indigenous woman, explained a specific instance where she attempted to save her partner during an overdose, despite anticipating her own risk of overdose:

*We both almost died, right? But I had to call an ambulance and it was, like, pure adrenalin that got me down four flights of stairs to go use the phone. And then back up again to go be with my husband until they [paramedics] came… He was foaming at the mouth in the corners. And he’s, like, “Get help. Get help.” And… and I was, like, “Get help? You want, like, an ambulance?” ’Cause he hates ambulances. So, I was, like, I wanted to make sure that he wanted an ambulance. And he’s, like, “Yeah, yeah. Help, help, help.” And so, I fucking… I was, like, running down the stairs and then, like, my legs started feeling, like, jelly underneath me. They were just, like, flying down the stairs, and, like, that’s when it hit me. Like, okay, that’s happening to him, it’s gonna happen to me, I took the same stuff.*

What is noteworthy about Esme’s experience is the level of labour she invested into ensuring her partner received the necessary care he required. Furthermore, Esme did this while cognizant of the fact that she would soon succumb to an overdose as well. Her actions exemplify the ways in which many participants practised overdose assistance in intimate partnerships, while simultaneously managing the risks this posed to their own health and safety.

“Lottie”, a 43-year-old white woman, described a similar experience with her partner, noting that she stayed with her ex-boyfriend during his overdose, despite her fear of arrest over outstanding warrants:

*My ex-boyfriend overdosed about a week and a half ago… Yeah. He said, “I might go under”, and then he went under. But I had warrants so I stood up and I was screaming for Narcan. And then a cop ran in, and as soon as the cop got to him, I left… My ex… my ex should have known. He should have never put me in that predicament. Like, because he knew I had warrants…*

While the Good Samaritan Drug Overdose Act exempts individuals from possession or condition violation charges, provided that they are seeking assistance for themselves or others [44], perceived risks remain. Lottie and Esme’s experiences reveal the ways women in this study may care for their partners regardless of the perceived risks to themselves. However, some participants expressed resentment over the level of care they were expected to provide for their partners.

Expressions of caregiver fatigue, stress, and frustration were commonly discussed at the OPS and were also reoccurring themes among participant accounts. Women in this study explained that they were expected to provide a certain level of (unreciprocated) care while simultaneously managing their own drug use. Mae was transparent about the how caring for her partner impacted her:

*I take care of him a lot [when he’s using drugs]. I make sure he doesn’t do anything… Yeah, it’s mostly a burden on me and I feel the whole thing on me. That’s why I feel depressed, because the whole thing’s on me. He figures he has it easy but it’s not. I’m having a hard… hard time.*

Mae revealed that the impact of repeatedly responding to her partner’s overdoses was “crazy”, and explained that the level of care she invested was connected to her depression.

Similarly, Lottie described caring for her overdosing partner as she simultaneously navigated her own drug use and overdose risk,

*when the fentanyl first came out and he would go under really quick. But he wouldn’t overdose. Like he blacks out and he doesn’t remember anything. But he would… he’d come to and be like, “How long was I out for?” Like he’d know. It was weird. Yeah. You’d think I was dealing with a child, like a baby in his body… It was brutal. Yeah, because I’d have to babysit him [while I was also using drugs at the same time] for like two hours till he’d snap out of it.*

Lottie explained that the care she was expected to provide was comparable to that which a person would invest in an infant. This comparison was shared by “Mireya”, a 40-year-old white woman, when asked if anyone relied on her for overdose prevention:

My ex does. He does that a lot. I’ve Narcanned him three times already in, like, three months. […] It’s like I’m babysitting every time I’m with him.

Mireya and other women’s accounts illustrate that WWUD who provide often unreciprocated overdose-related care in high-risk and informal settings, while navigating their own drug use and investing high levels of labour into their social circles, contend with feelings of burnout and negative impacts to their mental and emotional well-being.

#### Informal care: assisted injection

Assisted injection is an example of an act of care that extends beyond conventional public health conceptions of harm reduction. Several women reported that they had at one point or another assisted someone who had difficulty injecting themself:

*[I assisted with injections but] I don’t think I like the idea that I’m helping someone get high. But I do that anyway when I’m giving somebody a dragon [drug sharing through inhalation], too, so […]. Yeah. Like, I’d want somebody to do that for me, right?* (“Julie”, 33-year-old Indigenous woman)

*Um, like there was a few girls that I am comfortable with that do ask me if I can help them [with injecting]. But I’ve never, I’m not the one to ask anybody for help with that cause I do it myself… But there’s, like I said there’s a few girls I know that would ask me for help if I was there, ’cause they know me to help them, right.* (Marisol)

Participants shared that the need for assisted injection among WWUD was significant. Throughout the study period, we witnessed multiple requests for injection support as well as offers of such support by women at the OPS who, while acknowledging the risks, also characterized assisted injection as potentially life-saving for those women most in need.

According to study participants, decisions to provide assistance with injection were dependent on women’s relationships with one another: they only administered injections for those that they considered themselves close to or had a relationship with. “Yasmin”, a 42-year-old white woman, explained that she regularly aided with injections due to considerable demand amongst WWUD:

*Because a lot of people need help getting fixed [injected] because they don’t know how to do it… It’s hard for them to do that and a lot of girls, you know, don’t know how to do it and they can hurt themselves really bad doing that. So, I’m pretty experienced at that. I’ve never hurt anybody.*

Although participants’ decisions to practise assisted injection were influenced by various factors, many appeared to act in accordance with similar ethics of care. Julie and Marisol (quoted above) described how their discomfort with assisted injection was superseded by the significant need among those who require support with it and stated that this service was especially vital for women. Yasmin explained her decision-making was influenced not only by financial gain in some cases but also a desire to mitigate drug-related harms for women who inject drugs.

“Paige”, a 34-year-old white woman, described the expertise she draws on to support other women in need through assisted injection:

*We created those services [SisterSpace]. We weren’t allowed to use in the common room in our building, so a lot of girls couldn’t hit [inject] themselves; they wanted somebody to jug them, which is inject in their neck, and they couldn’t hit themselves. If you couldn’t doctor [assist with injecting] their neck, some people don’t want to do it because it’s scary to them. I have a pretty steady hand and a good technique because I learned from my friend who is a veterinary assistant how to do the injection properly, so I don’t have a hard time and usually get it the first poke*

Paige’s response exemplifies the ways in which informal care, performed by WWUD, can precede and eventually evolve into formal avenues (e.g. assistance in OPS) through which women provide and access harm reduction. Participants created spaces for WWUD to safely access forms of harm reduction care that remain absent or prohibited from many sanctioned and regulated drug-use services. However, several participants shared that, despite the considerable need for assisted injection, they were at times reluctant to engage in this practice.

For women in our study, risk perceptions influenced their decision-making around assisted injection. Rose stated that the potential consequences injecting other women can have, for herself and others, tempered her willingness to administer injections: “I don’t want to go to jail for something when I was just helping. So I try… no, I try to say no”. Similarly, “Tye”, a 52-year-old Indigenous woman, described her awareness and fear of the potential risks that came with assisted injection and cited them as the reason she no longer assisted others:

*People used to chase me around. Fuck, I was like a nurse. “[Tye] can you fix me?” ’Cause I was really... Now I just say no. I don’t say too much ’cause I don’t mainline anything. I think I’m more scared ’cause I don’t want somebody to die or OD… I... oh man, I don’t know how many people have come up to me in that place and say, “[Tye] can you jug me?” and I say, “Oh fuck, I haven’t done that in years.”*

Participants explained that they would hold themselves responsible for someone overdosing, and felt fearful that they could potentially be at fault for another person’s death. Their feelings towards assisted injection are noteworthy, as the level of concern participants express subverts pervasive stereotypes of WWUD as self-serving and incapable of caring for others. Instead, women who are capable of performing assisted injection may experience an increased burden of care.

## Discussion

This study, focused on WWUD’s experiences of engaging in harm reduction care while navigating their own drug use, demonstrates that for WWUD the boundary between formal and informal harm reduction care can be fluid. Participants described taking pride in their formal harm reduction work. However, engagement in harm reduction care was rarely limited to formal spaces. Instead, care was practised by women outside of the confines of regulated consumption spaces and guided by their own ethical codes.

Overdose care was a prominent practice performed by women. However, participants expressed that repeated exposures to overdose events negatively affected their mental and emotional health in an environment where people who use drugs have limited access to supportive services and resources. While there was a significant need for injection assistance among WWUD, fulfilled by many study participants, the provision of assisted injection was mediated by a fear of potential overdosing and associated legal sanctions.

Canada’s legacy of colonialism, slavery, and segregation manifests in persistent race-based inequities throughout the justice system, health care, and social services [39, 45, 46]. In Canada, Indigenous and Black women face disproportionately higher federal incarceration rates than their non-Indigenous and Black peers [47] and harsher consequences for using or possessing drugs [15, 39, 48]. Thus, fears regarding punitive responses to informal overdose prevention and care, including assisted injection, are not groundless.

Women in this study illustrated that providing harm reduction care required them to understand the needs of the communities they served (i.e. formal and informal overdose prevention), but in certain cases, it was necessary to also have relationships with the care receiver (i.e. assisted injection). If acts of care should be informed by the relational contexts in which they are performed [18], our study positions analyses of harm reduction care performed within interpersonal and community relationships in a society regulated by drug-prohibitionist laws. As such, WWUD’s provision of harm reduction care, while focused on meeting the substantial needs of their communities, was constrained by laws and policies that increase their risk of police interactions and limit their access to resources.

Incorporating a feminist care ethics approach allows for an understanding of the ways in which acts of harm reduction care performed by women are governed by such power dynamics [20], the relationship between care provider and care receiver [19, 21], and their willingness to be flexible and “work outside ‘the rules of the system’” [22], p. 11) (i.e. institutions and policies) in order to meet the needs of their communities. Power dynamics and relationships have played, and continue to play, a role in drug-use practices and harm reduction care delivery [49]. Current research indicates that for WWUD, relationship building is vital to the quality of care they receive from peer workers and that a shared experience can play a central role in facilitating this relationship building [50]. This is further supported by studies that demonstrate the importance of having people with lived experience involved in harm reduction care [7, 10, 50, 51], and the recognition that harm reduction care is diverse and extends beyond traditional normative concepts [9]. However, as evident in this study, these factors, as well as their influence on harm reduction and drug-use practices, may lead to increased burdens of care for women who use drugs.

Although women in this study discussed engaging in several types of harm reduction care, the most prominent was overdose-related care. Women’s experiences with care illustrated that the boundary between formal and informal harm reduction care, especially as it pertained to overdose prevention and reversal, was fluid and not restricted to formal harm reduction spaces. Engagement in overdose reversal by people who use drugs is well-documented by past research that describes the extensive ﻿level of labour invested in attending to overdose events, inside and outside of formal work, as well as the consequences exposure to regular overdose events has on one’s mental and emotional well-being [6, 7, 9, 10, 52]. While women in this study discussed the personal benefits they gained from engaging in overdose care, those benefits were complicated by feelings of burnout, anxiety over potential police interactions, and their own overdose risks. These complications were especially relevant for women who invested high levels of overdose care into their intimate partnerships. Statistically, women in heterosexual relationships are more likely to consume drugs after their partners (be second on the needle) [53]. With an increasingly toxic illicit drug supply, and a lack of widespread access to pharmaceutical-grade drugs, similar situations beyond our study context may not be rare. Resources such as non-stigmatizing access to pharmaceutical-grade drugs (e.g. a safer supply) [54] would be beneficial in addressing issues of overdose and the potential compounding effect it has on the health of WWUD who engage in overdose care, as it would allow people who use drugs to gauge the potency of their drug(s) of choice and limit their exposure to a toxic drug supply.

Previous research documents that a lack of access to assisted injection leaves women at risk of increased violence and disease transmission [27] and overdose risk [27, 55]. WWUD are more likely to need assistance injecting [27, 55], depend on partners to do so [27], and to experience gendered and racialized violence when accessing assisted injection in precarious environments [27, 30]. The policies that restrict assisted injection in formal harm reduction spaces create institutional-level barriers that lead to the exclusion of already vulnerable populations [26, 56] and disproportionately impact WWUD [27, 55]. This study illustrates how WWUD’s engagement in informal care fills voids in formal harm reduction care created by these systemic and institutional barriers.

Women in this study explained that their engagement in assisted injection was mediated by monetary incentives (in the context of income insecurity), their interpersonal relationships, their own “ethos of care”, and a sense of personal responsibility. Although women’s practice of assisted injection in unregulated environments is significant, women who access assisted injection in unsafe or informal environments can be at increased overdose risk [27]. Though Canada has lifted the ban on peer injection assistance within SCS since this study was conducted, it is not currently available at all sites [57]. Emerging research has explored the implementation of assisted injection within SCS, further verifying the importance of relationships in injection assistance and that WWUD disproportionately require assistance [58]. Given these factors, we argue for a wider implementation of peer assistance in formal harm reduction spaces alongside significant increases in health and social supports and ongoing consultation and meaningful collaboration with, and direction from, diverse people who use drugs. More broadly, the significant harm reduction potential of community-based existent and emergent non-normative modes of care must be acknowledged and prioritized [14].

While harm reduction, health care, and social services are meant to be accessible to all who require them, research has established that WWUD experience unique institutional and socio-structural barriers that can interfere with access (e.g. gendered and racialized stigma and violence) [59, 60]. This study illustrates the ways in which WWUD help bridge gaps in harm reduction care, such as overdose prevention and assisted injection, while simultaneously navigating a lack of access to necessary services, thereby exhibiting significant levels of resilience. Although harm reduction care provision is not exclusive to WWUD [7, 9, 10], their engagement in acts of harm reduction care under drug prohibition is compounded by issues of gender and racial inequality. Women who use drugs must contend with gendered norms of care and disproportionate participation in unpaid labour [34], sexual, racialized, and gendered violence [61], and the feminization of poverty and fewer economic opportunities [35, 62, 63]. For Black, Indigenous, and racialized women these inequities are intensified by the intersections of colonialism and institutionalized racism [39, 64], which contribute to increased incidences of violence [65], poorer health outcomes [46] and increased surveillance by and interactions with law enforcement and incarceration [39, 47]. Thus, the level of care WWUD provide within their interpersonal relationships has tangible negative consequences for their health and well-being, with intersecting axes of oppression exacerbating these consequences for Black, Indigenous, and racialized women who use drugs.

This study has several limitations. First, the data were collected from a limited sample size, and recruitment was limited to a specific geographic location. Thus, topics may not be generalizable; however, the findings do have implications for considerations of gendered ethics of harm reduction care within Canada as well as internationally. Secondly, study participants may have been inclined to give what they believed to be socially acceptable responses that they felt aligned with the researchers’ interests. Consequently, these study results are not representative of all women who use drugs. Additionally, this study was not originally designed to explore WWUD’s engagement in informal and formal care; rather, these themes came up organically over the course of discussion. As a result, the specific experiences of Indigenous, Black, and racialized WWUD, and the ways colonial histories, racism, and gender intersect and influence experiences of harm reduction care, were not thoroughly analysed [66–69]. Further, research may be necessary to fully explore the acts of harm reduction care WWUD regularly engage in the specific experiences of racialized women, transgender women, and non-binary people and examine the potential consequences of their engagement in said care.

## Conclusion

This study illustrates that WWUD are formally and informally relied upon by community members, intimate partners, and individuals within their social circles for harm reduction care. Women in this study discussed their engagement in care while simultaneously navigating institutional and systemic barriers, as well as their own drug use and the associated risks. This study highlights that harm reduction care is diverse, as documented by previous research, and mediated by multiple factors, including the nature of their relationship with the care receiver, monetary incentives, or a desire to give back and support one’s community. While women who provided harm reduction care derived positive emotions from the care they provided for others, they also reported experiencing caregiver burnout, impacts to their emotional and mental health, and other harms associated with the criminalization of drugs. Thus, existing gendered dynamics of care may put WWUD at an increased risk of a number of stressors negatively impacting their well-being. These study findings serve to subvert prevailing stigmatizing narratives of WWUD as self-interested or uncaring, and instead make evident the level of care and labour WWUD invest into their immediate communities and interpersonal relationships in the absence of institutional support and resources. However, further research is needed to fully understand the consequences of WWUD’s engagement in harm reduction care, in order to better support them and the communities they care for. It is necessary for drug-use policies and services to understand that as harm reduction care enters the public sphere (i.e. overdose reversal, consumption supervision, assisted injection) barriers that impact WWUD’s emotional, physical, mental, and financial well-being must be recognized and addressed.

## Data Availability

Not applicable.

## Abbreviations

OPS: Overdose prevention sites
SCS: Supervised consumption sites
PWLE: People with lived/living experience of substance use
WWUD: Women who use drugs
